# The Challenges and Possibilities of Reducing Deaths from Cryptococcal Meningitis in Sub-Saharan Africa

**DOI:** 10.1371/journal.pmed.1001318

**Published:** 2012-09-25

**Authors:** Andrew W. K. Farlow

**Affiliations:** Spatial Ecology and Epidemiology Group, Department of Zoology, University of Oxford, Oxford, United Kingdom

## Abstract

Andrew Farlow reflects on new research by David Boulware and colleagues, highlighting current challenges in treating cryptoccocal meningitis and the relative neglect of investment in research to treat this disease.

**Linked Research Article**

This Perspective discusses the following new study published in *PLOS Medicine*:

Rajasingham R, Rolfes MA, Birkenkamp KE, Meya DB, Boulware DR (2012) Cryptococcal Meningitis Treatment Strategies in Resource-Limited Settings: A Cost-Effectiveness Analysis. PLoS Med 9(9): e1001316. doi:10.1371/journal.pmed.1001316 http://www.plosmedicine.org/article/info:doi/10.1371/journal.pmed.1001316

David Boulware and colleagues assess the cost-effectiveness of different treatment strategies in low- and middle-income countries for cryptococcal meningitis, one of the most common opportunistic infections of people with HIV.

With increased access to life-saving drugs, HIV is becoming less of a death sentence in developing countries. But death may still occur as a result of opportunistic infections, one of the most common being cryptococcal meningitis (CM), a fungal infection of the brain and spinal cord that primarily affects people with weakened immune systems. Of the estimated 960,000 people each year infected with CM worldwide—about three-quarters in sub-Saharan Africa—approximately 625,000 (65%) die within 3 months of infection [1]. In this issue of *PLOS Medicine*, Radha Rajasingham and colleagues argue that, for relatively little cost and with drugs that already exist, about 150,000 more CM deaths could be prevented every year in sub-Saharan Africa.

Using available trial data and a variety of cost evidence, the authors calculate the cost-effectiveness of six CM induction treatment regimes under resource-limited conditions. Two results stand out. First, the least cost-effective regimen is high-dose fluconazole monotherapy, which has been associated with a nearly 2-fold higher 10-week mortality rate and about 30% greater absolute mortality than the short-course (5–7 days) amphotericin-based regimen. Second, long-course (14 days) amphotericin-based regimens, as recommended by the World Health Organization (WHO), are not only more costly but appear to be no more effective than short-course amphotericin-based regimens. If the second finding is confirmed in further trials, the WHO will need to adjust its advice.

## Doing Nothing Is Costly, Change Is Difficult, and Inertia Is Harmful

The authors provide an instructive lesson on the strengths and weaknesses of the use of cost-effectiveness analysis in policy-making. All six CM regimens generate costs per quality-adjusted life-year (QALY) gained that are well below the level used by the WHO to define an intervention as “highly cost-effective” [2],[3]. In the case of CM, doing nothing is very costly, so even though 60% (95% CI 54%–66%) of patients die within a year of fluconazole monotherapy, given its low cost it is still deemed “highly cost-effective”. The WHO threshold might be necessary, but it is certainly not sufficient.

We are reminded also of the importance of the specifics of health systems. Demonstrating that one treatment regimen dominates another does not mean that change will automatically follow. Many clinicians in sub-Saharan Africa rely on fluconazole monotherapy because the drugs are accessible, cheap, or even free, and do not require the laboratory monitoring necessary for the proper functioning of amphotericin. The authors are careful to use only data taken from resource-limited settings, but even this presupposes a health system functional enough to generate such data. In practice: manometers for safe and accurate control of intracranial pressure are often not available, necessitating improvisation; supply chains for safe lumbar punctures aren't always reliable; the use of IV fluids is sometimes erratic; and health workers are thin on the ground. Health systems come with budgets too. One treatment regimen might be superior to others in terms of QALYs gained, but QALYs accumulate over many years, whereas a change in practice may come up against a hard budget constraint in the here and now; the short-course amphotericin-based regimen achieves the lowest cost per QALY gained because of its superior survival rates, but it is still US$60 more expensive per patient than fluconazole monotherapy.

Awkward questions also arise regarding the potentially distorting impact of drug donation programmes, an issue that the WHO has grappled with for many years. Given that fluconazole monotherapy is “highly cost-effective”, the relative inertia of policy-makers and funders in making other drugs available may have inadvertently made it the only regimen available in many resource-poor settings. The evidence suggests that, if used, fluconazole needs at least to be combined with amphotericin if reliance on it is not to be detrimental.

## Proposed Research Strategy

The small size of some of the trials used by Rajasingham and colleagues may make readers uneasy, but it doesn't undermine their study—it highlights the relative neglect of this area of research. For the past decade or more, every year public, private, and philanthropic stakeholders have ploughed about US$1 billion into researching and developing HIV vaccines, microbicides, and drugs. So far, no life has been saved by a HIV vaccine or a microbicide. New drugs for CM are needed, but mitigating the tragedy of HIV through better treatment of CM also involves the better application of existing drugs. And yet, the trial sizes being used to determine the best treatment regimens are minuscule. If the results of this study hold up in further trials, the use of long-course amphotericin-based treatment—expensive and difficult to sustain in resource-limited settings—could be minimized, and a billion dollars spread over 30 or so years supporting short-course amphotericin-based treatment could buy nearly 5 million lives in sub-Saharan Africa. By any threshold, this would be highly cost-effective. If amphotericin became more affordable, the cost would be lower still.

It is difficult for risk-averse policy-makers to change their advice on the basis of comparisons involving underpowered evidence; the confidence intervals for mortality for all CM treatment regimes, but especially for those involving short-course amphotericin (e.g., 10-week mortality 95% CI: 19%–35%), are still too wide (and therefore sometimes overlapping) for comfort. This indicates, in particular, the need for bigger 5–7 day trials of amphotericin, including on its own (the authors are forced to lump together all 5–7 day data to achieve enough statistical power). Some head-to-head trials are in the pipeline (funded by the National Institutes of Health/AIDS Clinical Trials Group and the Medical Research Council), but the results are still some years away. It would be useful also to unravel the specific ways in which resource-limited settings impact upon survival rates. The lower efficacy of long-course amphotericin—and the logic therefore of ending treatment earlier—is related to the challenges in resource-limited settings of managing the toxicity that only really becomes a problem after most of the clearance of fungi has already been achieved. Low survival rates are also partly due to treatment often being delayed, to when cryptococcosis is more advanced [4]. Therefore, trials need to include routine screening and preemptive treatment.

The results of Rajasingham and colleagues suggest that spending modest levels of resources gathering better evidence now—together with a policy-making process capable of overcoming the status quo, and with funders willing to reconfigure their spending habits—may yield large benefits in terms of costs and lives saved in the future.

## Abbreviations

CM: cryptococcal meningitis
QALY: quality-adjusted life-year
WHO: World Health Organization
